# ASSOCIATION BETWEEN SOCIAL CAPITAL AND SUCCESSFUL AGING AMONG EMPTY NESTERS: A CROSS-SECTIONAL STUDY

**DOI:** 10.1093/geroni/igae098.2885

**Published:** 2024-12-31

**Authors:** Hui Chang, Zhi-wen Wang

**Affiliations:** Guizhou Medical University, Guiyang, Guizhou, China (People’s Republic); Peking University Health Science Center, Beijing, Beijing, China (People’s Republic)

## Abstract

**Aims:**

To determine the mediating and chain mediating roles of a sense of coherence and health-promoting lifestyle between social capital and successful aging among empty nesters to explain the internal mechanisms underlying the relationship.

**Methods:**

A total of 452 community-based empty nesters were included using convenience sampling. Empty nesters were surveyed using the Successful Ageing Scale, Social Capital Scale, Sense of Coherence Scale, and Chinese version of the Health-Promotion Lifestyle Scale-II. Descriptive analyses and correlation analyses were conducted using SPSS25.0. Mplus 8.3 was employed to perform chain mediation analyses. For this study, we followed the Strengthening the Reporting of Observational Studies in Epidemiology (STROBE) guideline.

**Results:**

There was a positive correlation between social capital, a sense of coherence, a health-promoting lifestyle, and successful aging. Social capital has a direct positive effect on successful aging. Sense of coherence and health-promoting lifestyles partially mediate the relationship between social capital and successful aging. The sense of coherence and health-promoting lifestyles also have a chain mediating effect.

**Conclusion:**

The quality of aging for empty nesters can be improved from the perspective of social capital. Effective measures can also be taken to improve the sense of coherence so that they can maintain a healthy lifestyle and thus achieve successful aging.

